# Dysfunction of Inferior Parietal Lobule During Sensory Gating in Patients With Amnestic Mild Cognitive Impairment

**DOI:** 10.3389/fnagi.2020.00039

**Published:** 2020-02-25

**Authors:** Chia-Hsiung Cheng, Fu-Jung Hsiao, Yu-Wei Hsieh, Pei-Ning Wang

**Affiliations:** ^1^Department of Occupational Therapy, Graduate Institute of Behavioral Sciences, College of Medicine, Chang Gung University, Taoyuan, Taiwan; ^2^Healthy Aging Research Center, Chang Gung University, Taoyuan, Taiwan; ^3^Department of Psychiatry, Chang Gung Memorial Hospital, Linkou, Taiwan; ^4^Laboratory of Brain Imaging and Neural Dynamics (BIND Lab), Chang Gung University, Taoyuan, Taiwan; ^5^Brain Research Center, National Yang-Ming University, Taipei, Taiwan; ^6^Department of Physical Medicine and Rehabilitation, Chang Gung Memorial Hospital, Linkou, Taiwan; ^7^Division of General Neurology, Department of Neurological Institute, Taipei Veterans General Hospital, Taipei, Taiwan; ^8^Department of Neurology, National Yang-Ming University, Taipei, Taiwan

**Keywords:** sensory gating, mild cognitive impairment, aging, inhibitory control, magnetoencephalography (MEG)

## Abstract

Patients with amnestic mild cognitive impairment (aMCI) demonstrate significant cognitive deficits, especially in the memory aspect. The memory deficiency might be attributed to the difficulties in the inhibitory function to suppress redundant stimuli. Sensory gating (SG) refers to the attenuation of neural responses to the second identical stimulus in a paired-click paradigm, in which auditory stimuli are delivered in pairs with inter-stimulus intervals (ISI) of 500 ms and inter-pair intervals of 6–8 s. It is considered as an electrophysiological signal to reflect the brain’s automatic response to gate out repetitive sensory inputs. However, there has been no study systematically investigating SG function in aMCI patients. Thus, the present study used magnetoencephalography (MEG) to record neuromagnetic responses to a paired-click paradigm in 23 healthy controls (HC) and 26 aMCI patients. The Stimulus 2/Stimulus 1 (S2/S1) amplitude ratio was used to represent the SG function. Compared to HC, aMCI patients showed M50 SG deficits in the left inferior frontal gyrus (IFG) and right inferior parietal lobule (IPL). M100 SG defects were also observed in the right IPL. Based on the ROIs showing significant between-group SG differences, we found that a more deficient M50 SG function in the right IPL was associated with poorer performance in the immediate recall of Logic Memory (LM), Chinese Version Verbal Learning Test (CVVLT) and Digit Span Backward (DSB) Test. Furthermore, the M50 SG ratios of the right IPL together with the neuropsychological performance of LM and CVVLT demonstrated very good accuracy in the discrimination of aMCI from HC. In conclusion, compared to HC, aMCI patients showed a significant SG deficit in the right IPL, which was correlated with the auditory short-term memory function. We suggest the combination of SG in the right IPL, LM and CVVLT to be sensitive indicators to differentiate aMCI patients from HC.

## Introduction

Amnestic mild cognitive impairment (aMCI) is considered as an intermediate phase between normal aging and Alzheimer’s disease (AD; Feldman et al., 2004; Petersen et al., 2014). Despite the intact function of activities of daily living, patients with aMCI are characterized by declined performance on standardized cognitive tests, particularly in the aspects of learning and memory. Inhibitory function plays a vital role in the memory performance since successful encoding and/or consolidation requires not only enhancement of task-relevant representations, but also suppression of task-irrelevant representations (Hasher and Zacks, 1988; Gazzaley et al., 2008; Chadick et al., 2014). Using functional magnetic resonance imaging (MRI), it has been shown that older adults demonstrated a relatively preserved capacity in the cortical enhancement of task-relevant stimuli, while a significant deficit in the top-down inhibition of cortical activities related to task-irrelevant stimuli (Gazzaley et al., 2005). Such a top-down inhibitory deficit was also found to be correlated with working memory performance (Gazzaley et al., 2005, 2008). In addition to top-down inhibitory function, bottom-up inhibition is a more fundamental ability in the early-stage information processing and may have an impact on the subsequent cognitive operations.

Sensory gating (SG) refers to the ability of the brain to automatically (i.e., bottom-up) inhibit the responses to the repetitive or redundant sensory inputs (Boutros and Belger, 1999; Cheng et al., 2015, 2016a, 2018). It has been proposed to serve as a protective mechanism against sensory inundation in the central nervous system (Patterson et al., 2008; Earls et al., 2016). SG is typically assessed in a paired-click paradigm in which two identical auditory stimuli are presented with an inter-stimulus interval (ISI) of 500 ms and an inter-pair interval of 6–8 s. Quantitatively, the amplitude ratio of the second stimulus (S2) over the first stimulus (S1; S2/S1) is calculated to reflect the SG function. A lower ratio reflects better performance in inhibiting irrelevant information (Cheng et al., 2016b, 2017b). In the electrophysiological recordings of auditory evoked potentials (AEPs), P50 (or its magnetic counterpart, M50) and N100 (or its magnetic counterpart, M100) are the two major components to assess SG. Since paired-click paradigm is a well-established and solid method, a number of clinical investigations have been conducted in patients with schizophrenia, and the results suggested a significant SG deficit either in the prodromal (Hsieh et al., 2012; van Tricht et al., 2015), acute (Devrim-Uçok et al., 2008; Oranje et al., 2013), or chronic (Brockhaus-Dumke et al., 2008; Micoulaud-Franchi et al., 2015) stage.

There have been some studies examining the SG function in neurodegenerative diseases, including dementia (Jessen et al., 2001; Cancelli et al., 2006; Thomas et al., 2010; Cheng et al., 2012; Josef Golubic et al., 2017). For example, Thomas and colleagues, recruiting 19 patients with probable AD and 17 healthy older adults, have revealed a significant P50 SG deficit in AD patients than in control subjects (Thomas et al., 2010). This result was similar to earlier reports in which mild AD (Cancelli et al., 2006) and moderate AD (Jessen et al., 2001) were studied. Regarding the association of SG function and neuropsychological assessments, a higher SG ratio (i.e., poorer inhibitory function) was reported to correlate with a more deficient performance on working memory, verbal fluency, and global cognitive function when AD and healthy older subjects were pooled together (Thomas et al., 2010; Josef Golubic et al., 2017). However, other studies failed to detect such a relationship (Jessen et al., 2001; Cancelli et al., 2006). Although previous studies have shown a deficit of SG in AD patients, there is no study, to the best of our knowledge, systematically investigating SG function by using paired-stimulus paradigm in patients with aMCI.

Considering the methodological issue, all of the aforementioned studies have applied electroencephalography (EEG) to compare SG ratios between AD and healthy controls (HC). With the limitation of electrode number and different conductivities of structures, it is less possible for EEG to probe the SG function at the source level. Magnetoencephalography (MEG), in contrast, has a better spatial resolution than EEG (Hari et al., 2010; Baillet, 2017) and therefore possesses a greater potential to disentangle the neural substrates underlying SG deficits. In addition, compared to the focal source modeling, the minimum norm estimate (MNE) is a distributed source imaging method, which can display a number of activated sources even when they overlap in time (Hämäläinen and Ilmoniemi, 1994). Thus, MNE has been considered to be a preferred strategy when analyzing multi-source evoked responses (Lin et al., 2006).

To be more specific, the goals of the present study were 3-fold. First, we attempted to test whether M50 and M100 SG ratios at the cortical level would be higher (i.e., worse function) in the patients with aMCI than those in the healthy older controls. Second, we sought to examine whether the regions exhibiting SG deficits would be associated with deteriorated neuropsychological performance, particularly those related to auditory short-term memory function because the memory impairment is the major clinical manifestation in aMCI patients. Finally, in order to differentiate aMCI from normal aging at the individual level, we further examined whether the SG ratio or its combination with short-term memory tests could serve as good indicators.

## Materials and Methods

### Participants

A total of 23 community-dwelling elderly adults (nine males, mean age = 69.04 ± 1.77 years) were recruited as the HC group. A total of 26 aMCI patients (14 males, mean ages = 69.96 ± 1.78 years) were enrolled from the outpatient memory clinic of the Department of Neurology, Taipei Veterans General Hospital. Each subject was interviewed by the neurologist (P-NW) to obtain a clinical history and neuropsychological performance. MRI and laboratory examinations were used to rule out tumors, strokes, severe white matter diseases. All participants had no history of epilepsy, alcoholism, major psychiatric illness, poly-pharmacy, or other systematic diseases that potentially affect cognitive function. The aMCI patients fulfilled the Peterson criteria (Petersen et al., 1999). They had objective memory impairment, MMSE ≥24, normal basic daily living activities, and without dementia (Wang et al., 2014). All of the subjects also reported no hearing impairment and normal or corrected-to-normal vision. Most of them were right-handed (handedness >80%) as evaluated by the Edinburg Inventory (Oldfield, 1971).

The present study was approved by the Institutional Review Board of Taipei Veterans General Hospital (Taipei, Taiwan), and was performed in accordance with approved guidelines and regulations. All the participants gave written informed consent after detailed descriptions of experimental procedures.

### Neuropsychological Testing

All the studying subjects underwent thorough neuropsychological assessments, including: (1) Mini-Mental State Examination (MMSE), with the proposed cutoff score between HC and dementia of 23/24 (Kochhann et al., 2010); (2) Chinese Version Verbal Learning Test (CVVLT), in which the proposed cutoff point of total score between HC and dementia was 20/21 (Chang et al., 2010); (3) Logic Memory (LM) Test of Wechsler Memory Scale, which has been shown to be a sensitive measure for detecting MCI and AD (Rabin et al., 2009); (4) Boston Naming Test, whose normative data from geriatric performance has been established (Jefferson et al., 2007); (5) Rey-Osterrieth Complex Figure Test, with the proposed cutoff score delayed recall subscale between HC and MCI of 18/19 (Takayama, 2010); (6) Trail Making Test Part A and B, whose psychometric properties have also been established in Chinese version (Wei et al., 2018); (7) Digit Span Forward and Backward Test (Muangpaisan et al., 2010); and (8) Verbal Fluency Test, with the proposed cutoff score between HC and those with cognitive impairments (MCI and mild dementia) of 16/17 (Alegret et al., 2018). Apolipoprotein E ε4 (APOE 4) genotyping was also performed in all subjects. The detailed demographic and neuropsychological data were presented in Table 1.

**Table 1:** Demographic variables and neuropsychological measures (mean ± SEM).

	HC (*n* = 23)	aMCI (*n* = 26)	*P*-values
Sex (male/female)	9/14	14/12	0.30
Age (years)	69.04 ± 1.77	69.96 ± 1.78	0.72
Education (years)	13.04 ± 0.72	11.12 ± 0.77	0.08
APOE 4 (yes/no)	5/18	4/21^a^	0.72
MMSE	28.83 ± 0.22	28.35 ± 0.25	0.16
STM	2.48 ± 0.15	2.27 ± 0.13	0.30
CVVLT
Total	31.00 ± 0.73	25.88 ± 0.84	<0.001
Delayed	8.26 ± 0.19	6.46 ± 0.30	<0.001
WMS Logic memory
Immediate	15.78 ± 0.80	10.04 ± 0.78	<0.001
Delayed	14.96 ± 0.84	7.81 ± 0.77	<0.001
CFT
Copy	32.48 ± 0.52	31.69 ± 0.66	0.36
Immediate	25.15 ± 1.25	19.52 ± 1.46	0.006
Delayed	24.74 ± 1.34	18.38 ± 1.44	0.002
VFT-animal	19.26 ± 0.92	15.46 ± 1.01	0.008
BNT
Spontaneous	27.09 ± 0.54	26.85 ± 0.54	0.75
Semantic cues	0.39 ± 0.15	0.19 ± 0.10	0.26
Phonemic cues	1.52 ± 0.29	1.46 ± 0.31	0.89
Digit Span Test
Forward	8.39 ± 0.24	8.00 ± 0.21	0.23
Backward	5.61 ± 0.34	4.65 ± 0.27	0.03
Trail Making Test
Part A (s)	16.83 ± 3.49	12.92 ± 0.85	0.29
Part B (s)	36.74 ± 5.65	48.23 ± 5.48	0.15

*SEM, standard error of the mean; HC, healthy control; aMCI, amnestic mild cognitive impairment; STM, short-term memory; CVVLT, Chinese Version Verbal Learning Test; WMS-Logic memory, Wechsler Memory Scale-Logic memory; CFT, Rey-Osterrieth Complex Figure Test; VFT-animal, Verbal Fluency Test-animal; BNT, Boston Naming Test. ^a^The blood test from one aMCI patient was not valid*.

### MEG Recordings

During MEG recordings, a paired-stimulus paradigm was presented to the subjects by means of Presentation software (version 11.3, Neurobehavioral System Inc., Davis, CA, USA). Stimuli consisted of a series of pairs of identical click-like tones (800 Hz, ISI = 500 ms, inter-pair interval = 6 s) and were binaurally delivered at the intensity of 60–70 dB through plastic earphones. Subjects were instructed to watch a silent, emotionally-neutral movie with subtitles and to ignore the auditory stimuli.

AEFs were recorded with a whole-head 306-channel MEG (Vectorview, Elekta-Neuromag, Helsinki, Finland). The sampling rate and online bandpass filter were set at 1,000 Hz and (0.1, 200) Hz, respectively. The head position in relation to MEG sensors was measured by four head position indicators (HPIs) attached to known sites on the scalp. The sites of three fiducial points (i.e., nasion, left and right preauricular points) and scalp surface were localized with a 3-D digitizer to allow alignment of the MEG and MRI coordinate systems. Electrooculograms (EOGs) attached above the left orbit and below the right orbit were used to monitor eye movements. In addition, heartbeats were recorded by electrocardiograms (ECGs). At least 100 pairs were collected from each participant for further analysis.

### MEG Data Analysis

In order to reduce the artifacts originating inside the device and external interferences outside the sensors, we applied MaxFilter from the Neuromag software system (Taulu et al., 2004; Taulu and Simola, 2006). Furthermore, all the acquired raw data contaminated by eye blinks and heartbeats were removed by using signal space projections (SSP), with the default setting in the Brainstorm software (Tadel et al., 2011).

The averaged AEFs were then offline filtered with a bandpass (1, 30) Hz, with a 100-ms baseline correction. The M50 peak was defined as the maximal response between 30 and 80 ms after the stimulus onset, and the M100 peak was defined as the maximal response between 70 and 160 ms after the stimulus onset.

The source activities of neuromagnetic data were analyzed by using depth-weighted MNE (Hämäläinen and Ilmoniemi, 1994) implemented in the Brainstorm software. The forward problem of MEG measures was resolved by the overlapping-sphere model (Huang et al., 1999), which estimates the strength of electrical dipoles located at the cortical surfaces. The noise covariance in the source estimation was calculated directly from the recordings. For each participant, the cortical-constraint MNE was computed over a set of ~15,000 dipoles distributed over the cortical envelope. Based on the prior knowledge from literature and our grand-averaged MNE results, a cluster of 30 vertices of 4–5 cm^2^ were manually selected to define regions of interest (ROIs) for M50 and M100, including bilateral superior temporal gyrus (STG; Edgar et al., 2003; Cheng et al., 2017a), bilateral middle temporal gyrus (MTG; Boutros et al., 2013; Cheng et al., 2015), bilateral inferior frontal gyrus (IFG; Garcia-Rill et al., 2008; Bak et al., 2014), and bilateral inferior parietal lobule (IPL; Boutros et al., 2013; Cheng et al., 2015). Although these anatomical structures cover a relatively wide area of cortical surfaces, the maximal activation cluster of each ROI in response to S1 was used as the center of the scout for both M50 and M100 from each participant. This method allowed us to extract the largest amplitudes of M50 and M100 to calculate SG ratios in each ROI.

In order to obtain the MNE source maps with a better signal-to-noise ratio, the time-resolved magnitude of each dipole was normalized to its baseline, yielding *z*-score values at each cortical location. The *z* scores were rectified to produce absolute magnitude changes above baseline levels. The peak response to S1 and S2 were extracted from each participant at the identified ROIs, and the SG ratio was derived from S2/S1 in the M50 and M100 components.

### Statistical Analysis

All the data were presented as mean ± standard error of the mean (SEM). All variables included in the final analysis were normally distributed as verified by the Kolmogorov-Smirnov test (*Z* < 1.168, *p* > 0.131). The differences of SG ratios (M50 and M100) between HC and aMCI groups were compared by means of independent *t*-test in each identified ROI. Based on the ROIs with significant between-group differences, partial correlations, with age, gender and years of education as covariates, were used to further investigate the relationship between SG ratios and auditory short-term memory assessments, such as CVVLT, Digit Span Backward (DSB), and immediate recall of LM Test. Finally, we applied receiver operator characteristic (ROC) curve analysis to test if the SG ratio or its combination with auditory short-term memory tests could differentiae aMCI from HC. For the area under the curve (AUC), AUC between 0.5 and 0.7 was considered less accurate, AUC between 0.7 and 0.9 was considered moderately accurate, and AUC above 0.9 was considered very accurate (Greiner et al., 2000). A *p*-value < 0.05 (two-tailed) was considered to be statistically significant. In the correlational analysis, *p*-values were corrected for multiple comparisons by the *Benjamini and Hochberg approach* (Benjamini and Hochberg, 1995).

## Results

The two study groups did not significantly differ by age, gender, years of education, APOE 4 carrier distribution and MMSE scores. However, the patients with aMCI performed worse than HC in most of the neuropsychological tests, including CVVLT, LM Test of Wechsler Memory Scale, Rey-Osterrieth Complex Figure Test, Verbal Fluency Test, and DSB Test (Table 1).

**Figure 1:**
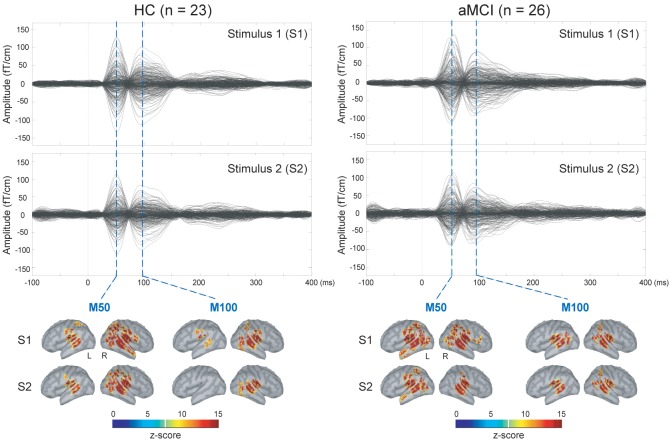
Upper panel: grand-averaged sensor waveforms of the auditory paired-stimulus paradigm in healthy controls (HC) and patients with amnestic mild cognitive impairment (aMCI). Lower panel: spatiotemporal dynamics of minimum norm estimate (MNE) regarding the M50 and M100 components. The cortical surfaces have been smoothed for better visualization (dark gray, sulci; light gray, gyri). L, left hemisphere; R, right hemisphere.

The upper panel of Figure 1 displays the grand-averaged AEFs to paired-click stimulation in the HC (*n* = 23) and aMCI (*n* = 26) groups. Compared to S1, neuromagnetic responses to S2 were reduced in both M50 and M100 components, either in the HC or aMCI subjects. The lower panel of Figure 1 shows the MNE source maps of M50 and M100 components. In addition to the temporal cortex, several regions of parietal and frontal cortices were activated to paired-click stimulation.

**Figure 2:**
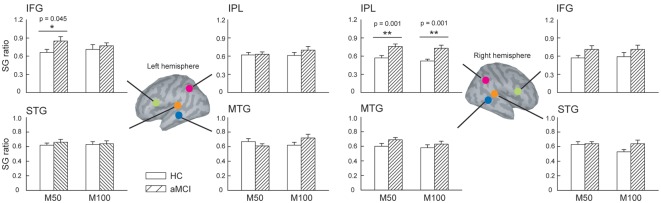
The regions of interest (ROIs) were manually identified in the bilateral superior temporal gyrus (STG), middle temporal gyrus (MTG), inferior frontal gyrus (IFG), and inferior parietal lobule (IPL) to study sensory gating (SG). Compared to HC, patients with aMCI demonstrated significantly higher M50 SG in the left IFG and right IPL. As for the M100 component, aMCI patients also showed an elevated SG ratio. These results suggest a deficit of inhibitory function in this clinical population. *p < 0.05, **p < 0.01.

We further compared the SG ratios between the two groups in the identified ROIs (Figure 2). There were no significant differences in the STG and MTG. However, we found that compared to HC, patients with aMCI demonstrated conspicuously higher M50 SG ratios in the left IFG (*t* = 2.063, *p* = 0.045) and right IPL (*t* = 3.726, *p* = 0.001). As for M100 SG, a significant between-group difference was also found in the right IPL (*t* = 3.550, *p* = 0.001).

**Figure 3:**
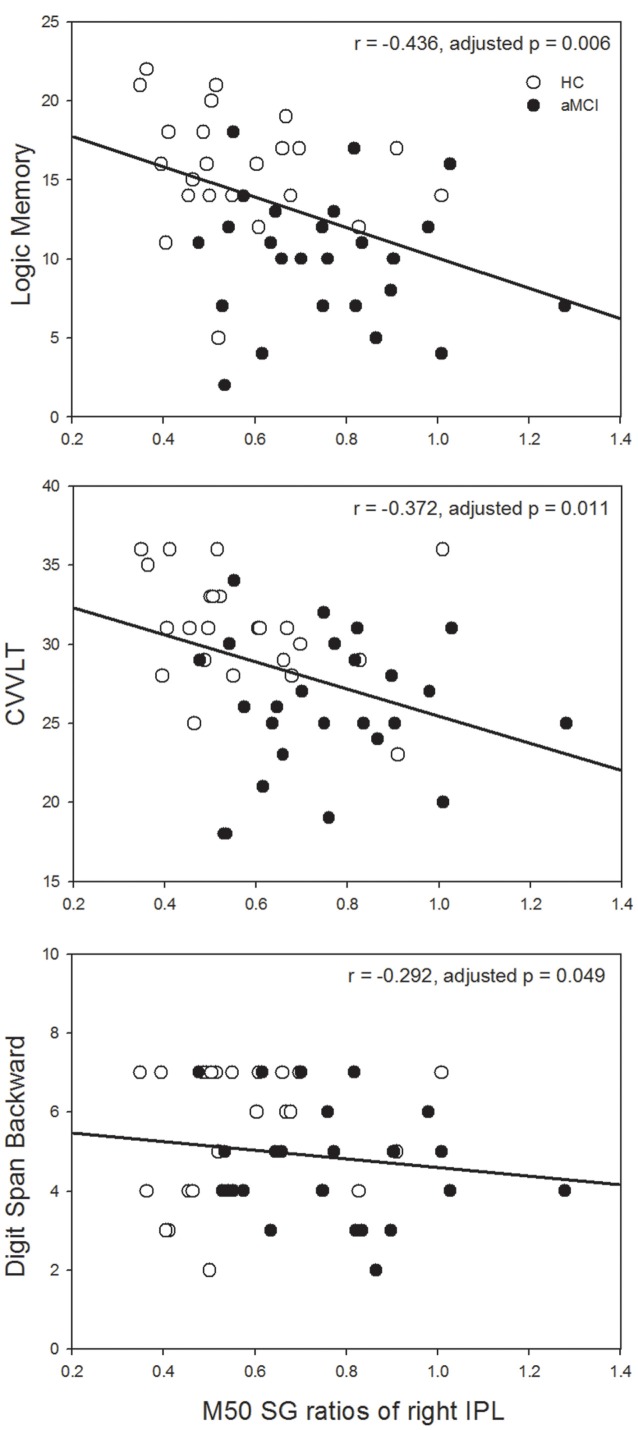
Higher M50 SG ratios in the right IPL were significantly associated with the worse performance of the Logic Memory (LM) Test (immediate recall), Chinese Version Verbal Learning Test (CVVLT), and Digit Span Backward (DSB) Test. HC, healthy control; aMCI, amnestic mild cognitive impairment.

Since the significant between-group differences were found in the left IFG and right IPL, we further investigated whether SG ratios in these ROIs would show associations with neuropsychological assessments in which auditory short-term memory function was involved. M50 SG ratios of the left IFG and M100 SG ratios of right IPL did not show any significant correlation with neuropsychological performance after the correction of multiple comparisons. M50 SG ratios in the right IPL were significantly correlated with scores of LM (immediate recall, *r* = −0.436, adjusted *p* = 0.006), CVVLT (*r* = −0.372, adjusted *p* = 0.011), and DSB (*r* = −0.292, adjusted *p* = 0.049; Figure 3).

**Figure 4:**
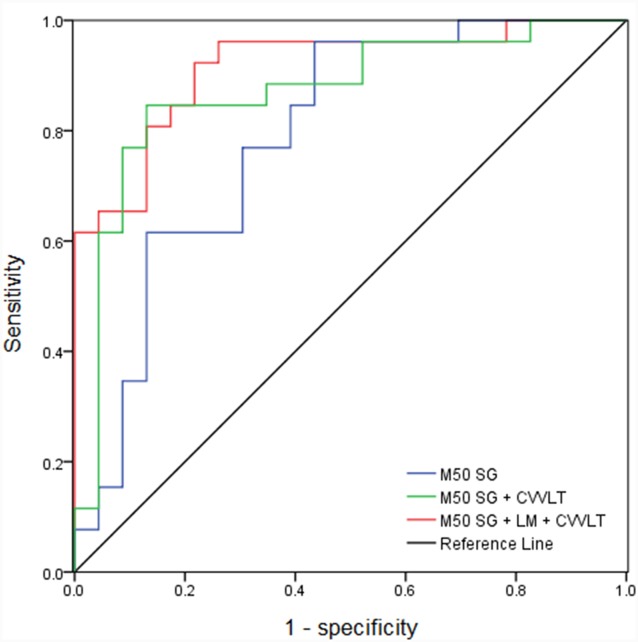
Receiver operator characteristic (ROC) curves of M50 SG ratio in the right IPL alone and its combination with scores of CVVLT and immediate recall of LM.

The AUC of M50 SG ratio of the right IPL was 0.791 (sensitivity = 0.846, specificity = 0.609), considered moderately accurate. Furthermore, this M50 SG ratio in combination with LM scores (AUC = 0.891, sensitivity = 0.885, specificity = 0.783) or CVVLT scores (AUC = 0.870, sensitivity = 0.846, specificity = 0.870) improved the discrimination ability (Figure 4). It was notable that the M50 SG ratio together with LM and CVVLT scores reached a very accurate ability in the discrimination of aMCI from HC (AUC = 0.915, sensitivity = 0.923, specificity = 0.783). Table 2 shows the detailed results of the ROC curve analysis.

**Table 2:** ROC curve analysis with the combination of different variables.

	AUC	Sensitivity	Specificity
M50 SG	0.791	0.846	0.609
M50 SG + LM	0.891	0.885	0.783
M50 SG + CVVLT	0.870	0.846	0.870
M50 SG + DSB	0.834	0.885	0.739
M50 SG + LM + CVVLT	0.915	0.923	0.783
M50 SG + LM + DSB	0.893	0.885	0.739
M50 SG + CVVLT + DSB	0.891	0.885	0.826
M50 SG + LM + CVVLT + DSB	0.916	0.885	0.826

*ROC, receiver operator characteristic; M50 SG, M50 sensory gating ratio in the right inferior parietal lobule; LM, Logic Memory; CVVLT, Chinese Version Verbal Learning Test, DSB, Digit Span Backward; AUC, area under the curve*.

## Discussion

This study compared the pre-attentive SG function between HC and aMCI at the source level and attempted to determine whether the SG ratios would be correlated with neuropsychological tests in which auditory short-term memory was involved. Our data yielded three major findings. First, compared to HC, patients with aMCI demonstrated deficient SG function in the left IFG and right IPL. Based on the aforementioned ROIs showing obvious between-group differences, M50 SG ratios of the right IPL were significantly correlated with the performance of short-term memory tests. Finally, ROC curve analysis revealed that the combination of the M50 SG ratio in the right IPL, LM and CVVLT had very good accuracy in differentiating aMCI from HC.

SG deficits have been evident in patients with AD (Jessen et al., 2001; Cancelli et al., 2006; Thomas et al., 2010; Cheng et al., 2012), whereas the relevant investigation on aMCI is extremely scarce. One of the major reasons is possibly due to the insensitivity of the recording instruments. In the previous EEG studies, midline electrodes, such as Fz or Cz, were analyzed to reflect the electrophysiological activities from the summation of temporal and frontal sources. Mastoid electrodes, on the other hand, were considered as a pure indicator of the temporal generators when the auditory evoked potentials were studied (Kujala and Näätänen, 2001; Cooper et al., 2006). However, the SG deficiency in the aMCI, compared to the control subjects, might not be obvious enough that can be detected by EEG electrodes. The advantage of MEG in its superior spatial resolution covers the weakness of EEG in the aspect of source localization. Our current MEG data, to some extent, supported this account since we did not find the significant between-group SG differences in the bilateral STG and MTG, both of which were considered as main neural generators of SG (Edgar et al., 2003; Cheng et al., 2017a). Instead, compared to the HC, aMCI showed a deficient SG function in the left IFG and right IPL. These findings suggested that reduced SG function in aMCI was attributed to the information processing deficits in the relatively higher-order centers (e.g., IFG and IPL), rather than basic sensory centers (e.g., STG and MTG).

Regarding the reduced M50 SG function of the IFG in aMCI patients, there are two plausible accounts to interpret our data. A prevailing contention is the inhibitory deficit hypothesis due to frontal dysfunction (Hasher and Zacks, 1988; Alain and Woods, 1999; Stothart and Kazanina, 2016). Compared to the younger adults, the elderly have been reported to demonstrate significantly larger P50 and/or N100 amplitudes to repetitive auditory stimulation (Chao and Knight, 1997). Furthermore, subjects with prefrontal damages showed an enhancement of P50 and/or N100 amplitudes to frequent auditory stimuli (Knight, 1984; Alho et al., 1994). Another account is the predictive coding hypothesis (Garrido et al., 2009; Grotheer and Kovács, 2016). SG or repetition suppression is an indicator of error minimization occurring when bottom-up sensory inputs from the level below (e.g., temporal cortex) coincide with the top-down predictions from the level above (e.g., frontal cortex; Friston, 2005; Auksztulewicz and Friston, 2016). Upon repetitive stimulation, the predictive error is reduced by adjusting synaptic activities within and between multiple hierarchical levels. By using a paired-stimulus paradigm, our data indicated that compared to the HC, aMCI patients exhibited higher SG ratios in the IFG, suggesting such inhibitory deficit may be indicative of a deficiency of top-down processing according to the predictive coding hypothesis.

The neurophysiological meanings regarding the reduced M50 and M100 SG of the IPL in aMCI patients remain extremely elucidative. First of all, it should be noted that the IPL is involved in the SG function. Using the grid and strip electrodes on the cerebral cortex, Boutros and colleagues have reported that in addition to the temporal lobe, the parietal cortex was part of neural circuits underlying P50 SG (Boutros et al., 2013) and N100 SG (Boutros et al., 2011). Our previous MEG study, by identifying a number of ROIs, has also found that S2-evoked M100 amplitude was significantly lower than S1-evoked M100 amplitude in the IPL among the younger adults (Cheng et al., 2015). The functional role of the IPL may be related to the monitoring of the information originating from other sensory cortex (Balslev et al., 2006; Schnell et al., 2007). In our previous MEG study, we did not observe age-related M100 SG differences in the IPL, suggesting healthy aging does not interfere with this function. However, when the pathological aging occurred, such as aMCI, SG was apparently deteriorated and could be detected at the basis of group comparisons. In addition, it should be noted that the AD-related pathologies occur a couple of years prior to the clinical manifestations. Also, the preclinical state can be longer than 2–3 years. Subjective cognitive decline (SCD), a self-perceived worsening in cognitive capacity along with normal performance on standardized cognitive assessments, has gained much attention over the past decade. It will be of clinical importance for future studies to investigate whether older adults with SCD show an altered auditory SG ability compared to those without SCD.

Based on the ROIs showing significant between-group SG differences, we further explored the relationships between SG and auditory short-term memory tests. Although there were several studies investigating the correlations between SG and all kinds of neuropsychological tests (Smith et al., 2010; Thomas et al., 2010; Hamilton et al., 2018), the present study only selected those in which auditory short-term memory was assessed since these cognitive assessments and SG were tested through the auditory modality. We found that M50 SG ratios of the right IPL were significantly correlated with the performance of short-term memory function (Figure 3). These results were consistent with previous studies showing that lower P50 SG and/or N100 SG ratios (i.e., better SG function) were related to better performance of attention and working memory in patients with schizophrenia (Smith et al., 2010; Hamilton et al., 2018). As for the AD patients, gating deficit has been related to the poor performance of DSB when healthy elderly and AD subjects were pooled together (Thomas et al., 2010). The novel result of the present study was that such an association was specifically observed in the IPL. Previous studies have applied regional homogeneity (ReHo) to measure local coherence of spontaneous brain activity and found that compared to HC, aMCI patients demonstrated reduced ReHo in the IPL (Zhang et al., 2012; Yuan et al., 2016). By analyzing the n-back working memory paradigm, a previous coordinate-based meta-analysis has shown that bilateral IPL was consistently activated across all the studies (Wang et al., 2019). In addition, a delicate study tracking cognitive changes over 6 months with longitudinal functional MRI data revealed a significant correlation between performance changes in free recall and brain activation changes in the IPL (McLaren et al., 2012). Taken together, our results suggest the critical role of IPL, particularly the right hemisphere, in the relationship between neurophysiological SG function and short-term memory performance.

We considered M50 SG in the right IPL as an acceptable neurophysiological indicator (AUC = 0.791, sensitivity = 0.846, specificity = 0.609) in differentiating aMCI from HC. A previous study applying another electrophysiological signal, called mismatch negativity (MMN), has revealed a similar accurate level as ours. More specifically, they found the MMN amplitude, but not latency, to be a reasonable biomarker (AUC = 0.76 for the first evaluation and AUC = 0.82 for the second evaluation; Lindín et al., 2013) for the discrimination between aMCI and middle-aged controls. In our present study, M50 SG ratios of the right IPL together with the LM and CVVLT further improved the discriminative accuracy. We suggest the combination of SG of the right IPL, LM and CVVLT to be sensitive indicators to differentiate aMCI patients from HC.

Conceptually, it is interesting to discuss the similarities/differences of the terminologies including SG and repetition priming (RP). Generally speaking, the aforementioned terms refer to the same phenomenon that the neural responses would be reduced after the repeated stimuli. However, based on different experimental paradigms or academic fields, there are somewhat different descriptions and meanings. The RP is usually studied with the semantic judgment task or working memory task, in which the subjects are required to respond to targets (Olichney et al., 2000, 2008; Yang et al., 2014; Broster et al., 2018). A stronger repetition effect (e.g., higher accuracy rate or shortened reaction time to the subsequently repeated stimuli, reduced amplitude after the repeated stimuli, etc.) indicates better memory-related performance since the N400 and/or P600 components are measured (Olichney et al., 2000, 2008; Yang et al., 2014). The SG is usually studied with the auditory (Smith et al., 2013; Rosburg, 2018) and somatosensory (Kisley and Cornwell, 2006; Cheng and Lin, 2013) paired-click paradigms, in which the subjects do not require to make a behavioral response. A lower ratio, that is more neural suppression to the repeated stimuli, indicates better SG function. Due to the independence of the behavioral requirement, SG has been widely studied in the clinical populations who have difficulties in maintaining attention and motivation. In our present study, aMCI and HC did not show significant differences of SG ratios in the primary sensory cortex (i.e., STG) but in the IPL, which was consistent with a previous report showing that patients with AD demonstrated spared repetition effect in the primary visual cortex (Broster et al., 2018). These findings also supported the prevailing notion that during neurodegenerative processes, most of the cortices (frontal cortices, parietal cortices, cingulate regions) apart from primary cortices have experienced major pathophysiological changes.

Several limitations of the present study must be acknowledged. First, the sample size was relatively small, which might impede us to find significant differences of APOE 4 carrier distribution between HC and aMCI. Previous large-scale studies have suggested that compared to the healthy older adults, the prevalence of APOE 4 was increased in patients with aMCI (van der Flier et al., 2008; Edmonds et al., 2015). Second, the individual’s hearing threshold was not collected in this study. Despite self-reportedly no obvious hearing impairments from our participants, we could not rule out the possibilities of hearing acuity on central auditory processes due to aging. However, it has been shown no significant age-related differences in hearing threshold at 1,000 Hz (Horváth et al., 2009). All of our subjects were older adults, which represented a more homogeneous sample in terms of auditory acuity. It was also important to note that the frequency we used in the present study was 800 Hz so that all the participants could successfully register the auditory inputs. Finally, the significant associations between neurophysiological function and neuropsychological performance did not allow us to infer their causality. Future research, which investigates whether the changes of SG will show concomitant changes along with the neuropsychological performance, is needed.

In conclusion, compared to HC, aMCI patients exhibited SG deficits, particularly in the right IPL. Such a deficiency was also related to the immediate recall of auditory memory tests. Our data further highlighted the importance of the combination of SG ratios and short-term memory tests in the discrimination between HC and aMCI.

## Data Availability Statement

All datasets generated for this study are included in the article.

## Ethics Statement

The studies involving human participants were reviewed and approved by Institutional Review Board of Taipei Veterans General Hospital (Taipei, Taiwan). The patients/participants provided their written informed consent to participate in this study.

## Author Contributions

C-HC and P-NW conceived and design the work and wrote the article. C-HC acquired the data. C-HC, F-JH, and Y-WH analyzed the data and participated in the discussion and provided the comments. All of the authors have read and approved the manuscript.

## Conflict of Interest

The authors declare that the research was conducted in the absence of any commercial or financial relationships that could be construed as a potential conflict of interest.
